# Patterns in deer-related traffic injuries over a decade: the Mayo clinic experience

**DOI:** 10.1186/1757-7241-18-46

**Published:** 2010-08-17

**Authors:** Dustin L Smoot, Martin D Zielinski, Daniel C Cullinane, Donald H Jenkins, Henry J Schiller, Mark D Sawyer

**Affiliations:** 1 Department of Surgery, Division of Trauma, Critical Care and General Surgery, Mayo Clinic, Rochester Minnesota, USA

## Abstract

**Background:**

Our American College of Surgeons Level 1 Trauma Center serves a rural population. As a result, there is a unique set of accidents that are not present in an urban environment such as deer related motor vehicle crashes (dMVC). We characterized injury patterns between motorcycle/all-terrain vehicles (MCC) and automobile (MVC) crashes related to dMVC (deer motor vehicle crash) with the hypotheses that MCC will present with higher Injury Severity Score (ISS) and that it would be related to whether the driver struck the deer or swerved.

**Methods:**

The records of 157 consecutive patients evaluated at our institution for injury related to dMVC from January 1^st^, 1997 to December 31^st^, 2006 were reviewed from our prospectively collected trauma database. Demographic, clinical, and crash specific parameters were abstracted. Injury severity was analyzed by the Abbreviated Injury Scale score for each body region as well as the overall Injury Severity Score (ISS).

**Results:**

Motorcycle crashes presented with a higher median ISS than MVCs (14 vs 5, p < 0.001). Median Abbreviated Injury Score (AIS) of the spine for MCC riders was higher (3 vs 0, p < 0.001) if they swerved rather than collided. Seventy-seven percent of riders were not wearing a helmet which did not result in a statistically significant increase in median ISS (16 vs 10), head AIS (2 vs 0) or spine AIS (0 vs 0).

Within the MVC group, there was no difference between swerving and hitting the deer in any AIS group. Forty-seven percent of drivers were not wearing seat belts which resulted in similar median ISS (6 vs 5) and AIS of all body regions.

**Conclusions:**

Motorcycle operators suffered higher ISS. There were no significant differences in median ISS if a driver involved in a deer-related motor vehicle crash swerved rather than collided, was helmeted, or restrained.

## Background

**Figure 1:**
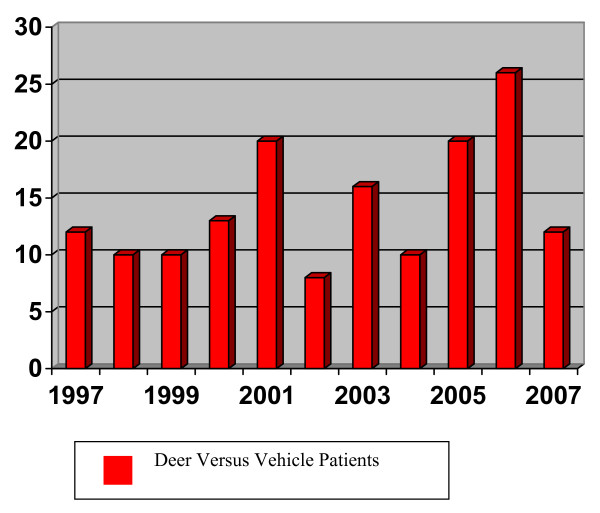
**Deer versus Vehicle Trauma Patients by Year-Mayo Clinic**.

Our American College of Surgeons Level 1 Trauma Center serves a rural population surrounding Rochester Minnesota. Our trauma catchment area extends roughly 50 miles radially encompassing parts of western Wisconsin, northern Iowa and south central Minnesota. As a result, there is a unique set of accidents that are not present in an urban environment such as deer related motor vehicle crashes (dMVC). State and national databases confirm a rising incidence of dMVC due to increasing mileage, encroachment on natural habitat and a larger deer herd (Figure 1). From 1997 to 2006, there were 45,421 reported dMVC in the state of Minnesota. This is likely an underestimation as the Department of Natural Resource (DNR) removed 98,054 carcasses from Minnesota roadways during this same timeframe [1,2].

Despite encroachment on deer habitat by human habitation and thinning of the herd through systematic hunting, the deer herd in Minnesota has actually grown. DNR pre-white tail hunt estimates placed the herd size at 733,000 in 1997, which ballooned to 1.2 million in 2006 [1,3]. This has resulted in increased likelihood of dMVC [4]. Insurance industry data suggest a driver in Minnesota now has a 1 in 156 chance of being involved in a dMVC where the national average is 1 in 208 [4].

A large push towards preventative strategies and driver education has been championed, but the medical literature lacks data analyzing crash characteristics [5-10]. We aimed to characterize the outcomes and injury patterns between motorcycle/all-terrain vehicles (MCC) and automobile (MVC) crashes related to dMVC with the hypotheses that MCC will present with higher Injury Severity Score (ISS) and that higher ISS would be directly related to whether the driver struck the deer or swerved.

## Methods

Following Institutional Review Board approval, the records of 157 consecutive patients evaluated at our institution for injury related to dMVC from January 1^st^, 1997 to December 31^st^, 2006 were reviewed from our prospectively collected trauma database. Crashes due to other animals were eliminated. Demographic, clinical, and crash specific parameters were abstracted. Injury severity was analyzed by the Abbreviated Injury Scale score for each body region as well as the overall Injury Severity Score (ISS). ISS > 15 defined severe trauma.

Descriptive statistics are reported as percentage for discrete variables with bivariate analysis of categorical variables using the Pearson's chi squared or Fisher's exact tests as indicated. Continuous variables were analyzed by the Wilcoxon rank sum test and reported as median with the corresponding range. Statistical significance was determined by *p *< 0.05.

## Results

Of the 157 patients identified, 116 (74%) were male. The median age for the group was 41 years (range 14-76). Automobiles were responsible for 54% of the dMVC, while a MCC was involved in the remaining 46% (Table 1). Specific injuries varied widely but the most common were closed head injuries and extremity fractures (Table 2).

**Table 1:** Demographics

	MVC	MCC
Age (mean)	14-74 (38)	15-76 (38)

Male (%)	58 (68%)	58 (81%)

ISS (median)	1-36 (5)	1-43 (14)

Etoh (# positive)	23 (27%)	8 (11%)

AIS (max/median) Head	5/0	5/0

AIS (max/median) Torso	4/0	4/0

AIS (max/median) Spine	3/0	3/0

AIS (max/median) Ext	3/0	3/0

**Table 2:** injury specifics

Injury Type	Number of patients
Intracranial Hemorrhage	20

Closed Head injury	80

Spinal Fracture	30

Pneumothorax/hemothorax	24

Rib Fracture	34

Liver injury	5

Spleen injury	6

Kidney injury	6

Extremity Fracture	60

Crash data revealed that vehicle operators swerved in 45% of the encounters and collided with the deer in 55%. Of the operators swerving, 6 were MCC and 64 MVC. The group that hit the deer directly included 66 motorcycles and 21 automobiles (Table 3). Motorcycle operators were more likely to collide with the deer (92% vs 8%, p < 0.001) while MVCs were more likely to swerve (75% vs 25%, p < 0.001). Motorcycle crashes presented with a higher median ISS than MVCs (14 vs 5, p < 0.002), but ISS was equivalent whether the patient swerved or collided in either vehicle type (MCC 16 vs 14, MVC 5 vs 5).

**Table 3:** crash data

	Motor vehicle (MVC)	Motorcycle (MCC)
**Total number crashes**	85	72

**Swerve mechanism**	64 (75%)	6 (8%)

**Hit mechanism**	21 (25%)	66 (92%)

**Helmet**	N/A	17 (23%)

**Restrained**	45 (53%)	N/A

Median Abbreviated Injury Score (AIS) of the spine for MCC riders was higher (3 vs 0, p < 0.001) if they swerved rather than collided, but there was no difference in head, torso or extremity injury severity. Seventy-seven percent of riders were not wearing a helmet which did not result in a statistically significant increase in median ISS (16 vs 10), head AIS (2 vs 0) or spine AIS (0 vs 0).

Within the MVC group, there was no difference between swerving and hitting the deer in any AIS group. Forty-seven percent of drivers were not wearing seat belts which resulted in similar median ISS (6 vs 5) and AIS of all body regions. There were 2 deaths, both in the MVC group (Table 4).

**Table 4:** Treatment and Outcomes

	MVC	MCC
**Number of Procedures**	67	159

**ICU admission**	26	37

**Discharge to home**	78	56

**Discharge to care facility**	5	16

**Death**	2	0

Alcohol (EtOH) intoxication was a factor in 31 of the crashes, 8 motorcycle and 23 automobile (Table 1). Twenty-five of the crashes involved drivers with EtOH levels exceeding the state legal limit of 0.08 with a mean of 0.13 (range 0.036-0.31). The median ISS was 9 (range 5-38) in the intoxicated motorcycle riders which was equivalent to the unimpaired riders (9, range 1-43). None of the intoxicated motorcycle riders were helmeted. Motor vehicle crashes involving EtOH had a median ISS of 9 (range 1-36); while non-EtOH related MVCs have a similar median ISS of 9 (range 1-34).

Time of day and season were also factors. Fifty-six percent of dMVCs happened from dusk until midnight (i.e. 1700 through 2400), with 80% happening at nighttime between 1700 and 0600. The majority of crashes happened in summer with 69 (44%); 44 MCC and 25 MVC. Summer and fall seasons accounted for 72% of the overall crashes.

## Discussion

Animal versus vehicle crashes are described throughout the literature. Pattern and severity of injury seems related to vehicle and animal size [5-7]. Abu-Zidan *et al *found Kangaroo-vehicle trauma in Australia resulted in a relatively mild pattern of injury with mostly head/face and extremity trauma. One patient suffered intracranial trauma and only one death occurred in their study [5]. Moose, on the other hand, result in far greater injury severity when involved in a vehicular crash. Farrell *et al *found an average ISS of 15.7 with a 9% mortality rate for their series. Head/face and cervical spine injuries predominated [6].

Deer are the predominate species in animal versus vehicle trauma in Minnesota and the upper Midwest. In Wisconsin, Nelson *et al *looked at motorcycle trauma as it related to white tail deer. Unhelmeted riders were found to have a higher ISS compared to their helmeted counterparts. The mortality and injury pattern was substantial with 7 deaths along with a predominance of head, chest and orthopedic injuries [7]. This is in contrast to our review which found no such association between helmet use and injury severity.

Our findings also contradict the most recent Cochrane database review from 2008. In pooled analysis, helmets were found to reduce the risk of death by 42% and the risk of head injury by 69% [11]. Our data sample is limited and may explain the concordant results. The question remains important however, with recent literature suggesting a significant economic impact as well between helmeted and unhelmeted riders estimated at $250,231,734 annually for hospital care [12].

For our series, MCC suffered, on average, higher ISS although the only deaths were in the MVC group. Despite our hypothesis, there were no significant differences in median ISS if a driver involved in a dMVC swerved rather than collided, was helmeted, or restrained. Spine injuries were more common in the swerve category of MCC, but injury pattern did not differ within the other groups.

Although severity differences were not appreciated, it is clear that deer versus vehicle accidents remain a serious clinical problem. During the nine years of our study, a total of 37 fatalities and 4221 injuries were recorded in the state of Minnesota [1,2]. Nationally, there is also a rising incidence. According to the National Highway Traffic Safety Administration Fatality Analysis Reporting System, an average of 111 fatal crashes involved animals between 1992 and 1995 increasing to 154 between 1998 and 2001 [13]. This trend will likely continue unless further strategies are implemented.

Financial concerns, while secondary to the human toll, are also great. The National Department of Transportation estimates that the average cost for an animal versus vehicle crash is $6,126 per incident [14]. This results in an estimate of over $1 billion annually for vehicle damage and medical expenses and does not take into account the incidents not reported [14]. Phone surveys have shown only half of animal versus vehicle crashes are reported to the police and less than half are reported to insurance companies [8]. Clearly cost is substantial and can be lowered if dMVCs can be reduced.

Our study is limited by the unknown number of vehicles that swerve to miss a deer and do not result in injury. This resulted in only 6 patients in the MCC swerve category that needed evaluation at our Trauma Center. This limits our ability to make general recommendations from the data set regarding preventative measures as they relate to vehicle type and avoidance strategies. We suspect that the low number of motorcyclists who swerved and required care at our facility indicate that a swerving maneuver on a motorcycle may be effective in avoiding injury. Unfortunately, we are unable to show this with the available data as we do not have an accurate denominator for comparison.

Increased driver vigilance during peak accident hours and seasons, as well as continued public education regarding the scope of the problem, may help decrease the number of incidents. More aggressive herd control also may help reduce dMVC. While increased hunting is considered the most effective method, other, non-lethal, techniques including fencing and reflectors, have also been used with mixed success [8]. The cost, however, can be prohibitive. Iowa estimates the cost of one mile of single-sided fencing to run $42,000 [8]. Although cheaper, the data on use of roadside reflectors is conflicting [9,10]. The State of Minnesota has not yet implemented structural changes to roadways to try to decrease deer-vehicle interactions.

## Conclusions

Deer-related motor vehicle crashes remain a costly mechanism from both a human and financial standpoint. While our study did not show a difference in median ISS based on crash mechanism, it did show increased trauma burden for motorcycle riders regardless of their helmet usage. Continued study of cost-effective preventative measures aimed at reducing the number of deer crossing motor ways appears to have the best chance of decreasing the spread of this rural menace.

## Competing interests

The authors declare that they have no competing interests.

## Authors' contributions

All authors have read and approved the final manuscript. Design of the study was performed by MDS, MDZ and DCC. Data collection and synthesis was completed by DLS and MDZ. Manuscript preparation was performed by HJS, DHJ, DLS and MDZ. Final proofing of the manuscript was by DCC, MDZ and DLS.
